# Pulp volume changes after piezocision-assisted tooth movement: a randomized clinical trial

**DOI:** 10.1186/s12903-020-01382-2

**Published:** 2021-01-13

**Authors:** Abdulkarim A. Hatrom, Mohammed S. Howait, Khalid H. Zawawi, Ghassan A. Al-Turki, Reem A. Alansari, Nouf F. Almehayawi, Sarah H. Alammari, Raghda A. Mohammed, Ali H. Hassan

**Affiliations:** 1 Department of Orthodontics, Alnoor Hospital, Ministry of Health, Makkah, Saudi Arabia; 2 Department of Endodontics, Faculty of Dentistry, King Abdul Aziz University, Jeddah, Saudi Arabia; 3 Department of Orthodontics, Faculty of Dentistry, King Abdulaziz University, Jeddah, Saudi Arabia; 4 Faculty of Dentistry, King Abdulaziz University, Jeddah, Saudi Arabia; 5 Taibah University, Madinah, Saudi Arabia; 6 Alfarabi Private College, PO Box 80209, Jeddah, 21589 Saudi Arabia

**Keywords:** Cone-beam computed tomography, Dental pulp, Dental pulp cavity, Orthodontic, Piezocision, Tooth movement

## Abstract

**Background:**

Orthodontic treatment may result in undesirable side effects, such as root resorption and a decrease in the size of the pulp tissue which could be associated with the duration of the orthodontic treatment. Piezocision-assisted tooth movement was introduced as a minimally invasive surgical procedure to shorten orthodontic treatment time. This prospective randomized clinical trial was aimed to compare the pulp volume changes of maxillary anterior teeth after en-masse retraction with or without piezocision-assisted orthodontics.

**Methods:**

Patients who required orthodontic treatment with bilateral maxillary first premolar extractions and en-masse retraction were recruited. Patients were randomly divided into extraction with piezocision, or only extraction, serving as controls. Pulp volume and root length changes of the maxillary six anterior teeth were measured and compared between the two groups using a 3-Dimensional analytical software. Paired and independent sample t-tests were used to compare within and between groups. Bivariate correlation was done between the mean change in pulp volume and its corresponding root length. The significance level was set at α = 0.05.

**Results:**

A total of 23 patients were included, 12 in the piezocision, and 11 in the control group. At the end of the en-masse retraction phase, (mean = 122.74 ± 3.06 days) pulp volume was significantly decreased in all six anterior teeth in both groups (*P* < 0.01). The decrease in pulp volume was not statistically different between both groups, (*P* > 0.05). There was a statistically significant but moderate correlation only between the pulp volume change of the right canine and its root length, r = 0.44, *P* = 0.034.

**Conclusions:**

The effect of piezocision-assisted orthodontic tooth movement on the pulp volume was comparable to the conventional orthodontic treatment. The degree of change in pulp volume does not appear to be related to the amount of root resorption.

*Trial registration* NCT03180151. Registered December 25, 2016, retrospectively registered, https://clinicaltrials.gov/ct2/show/record/NCT03180151.

## Background

During orthodontic tooth movement, tissues that are affected by orthodontic force application include the periodontal tissues, root length, and the pulp tissues [1–4]. The effect of orthodontic forces on pulp tissues has been the subject of interest with inconsistent results. Several studies have demonstrated a negative impact from orthodontic force application on the pulp tissues [3, 5–9]. While others did not find a relationship [10–12].

In an animal study, changes that occurred in the dental pulp as a result of orthodontic forces were observed in the form of pulpal aging and disruption of the odontoblastic layer at various locations [13]. Human pulpal response to orthodontic forces after 5 and 10 days showed a significant increase in angiogenesis and the degree of the pulpal response and was found to be dependent on the force applied [6]. These changes ranged from a minor alteration in the pulp vasculature to a decrease in respiration rate of the tissue, vacuolization of odontoblasts, apoptosis, alteration of alkaline phosphatase level and tissue damage aspiration [14]. When assessing the pulp size before and after orthodontic treatment with maxillary premolar extraction using cone-beam computed tomography (CBCT) found that the size of the pulp was significantly decreased following orthodontic treatment [8]. A recent study investigated the effect of forces applied by the rapid palatal expansion appliance on pulp volume [9]. They found a decrease in pulp volume at the end of the expansion.

The detection of pulp changes is commonly performed using periapical and cephalometric radiographs. However, these methods have inherited drawbacks such as magnification errors and reduced accuracy [15, 16]. Moreover, measuring the pulp cavity area using conventional 2-dimensional radiographs is not reliable. Histologic studies conducted to assess pulp changes due to orthodontic forces and tooth movement are accurate [14]. Unfortunately, this method can only be performed on extracted teeth, rendering it clinically inapplicable. Cone-beam computed tomographic (CBCT) imaging has modernized orthodontic diagnosis and treatment planning as well as being an accurate tool to detect root resorption with high sensitivity and specificity and enables accurate measurement of pulp volume [8, 9, 16, 17].

Piezocision-assisted orthodontic tooth movement was introduced as a more conservative surgical procedure to shorten orthodontic treatment time [18]. Thus far, the effect of piezocision-assisted en-masse tooth movement on pulp volume has never been investigated. Therefore, this study aimed to evaluate the changes in pulp volume after piezocision-assisted orthodontic tooth movement at the end of the en-masse retraction phase. A secondary aim was to evaluate the association between the change in pulp volume and root resorption.

## Methods

This parallel randomized clinical trial was approved by the Research Ethics Committee of the Faculty of Dentistry at King Abdulaziz University (No. 078–16) and the ethical standards of the Declaration of Helsinki. The research was registered under the Clinical Trial Registry (NCT03180151) and the updated CONSORT statement for reporting randomized clinical trials was followed.

The study sample was taken from a previous clinical trial [19]. Briefly, Orthodontic patients were recruited from the Faculty of Dentistry at King Abdulaziz University according to the following inclusion criteria: (1) patients age between 16–26 years, (2) half cusp or more Class II division 1 malocclusion, (3) mild or no crowding, (4) maxillary arches requiring bilateral first premolar extractions as part of the orthodontic treatment plan, and (5) good oral hygiene and healthy periodontal condition. The exclusion criteria were: (1) history of systemic disease, (2) presence of dental or facial anomalies, (3) patients receiving treatment that may affect bone biology and/or tooth movement, and (4) history of periodontal surgery. After obtaining informed consent, a simple randomization method was used as follows: patients were randomly assigned into two equal groups: (1) piezocision group (n = 13) and (2) control group (n = 13), using the opaque sealed envelope technique.

Sample size calculation was based on the post hoc power analyses using G Power[20] with effect size d = 2.03, power (1 − β) set at 0.95 and ⍺ = 0.05, two-tailed. The analysis showed that power reaches 0.916 when sample sizes are Piezocision group = 12 and Control group = 11 for group differences to reach statistical significance at the 0.05 level. This analysis showed that our sample size did not compromise the statistical power and was sufficient on effect size observed on the basis of the mean between-group comparison.

All patients were treated using a modified bidimensional bracket system 0.018-inch slots in the six anterior teeth and 0.022-inch slots in the posterior teeth (Gemini Series Bracket; 3 M Unitek, Monrovia, CA, USA). Also, all patients had miniscrews (3 M Unitek, Monrovia, Calif, USA) inserted bilaterally between the maxillary first molar and second premolar. After leveling and alignment, all patients had their upper first premolars extracted. In the piezocision group, a canine to canine piezocision decortication was performed only labially during the extraction procedure and no grafting was done. The en-masse retraction was started a week later for both groups on a 0.018 × 0.025-inch stainless steel arch-wire through nickel-titanium active coil springs delivering a bilateral 250grams of force. To monitor the force level, a strain caliper was used (Dentaurum, Ispringen, Germany).

During the study, one patient from the piezocision group and two patients from the control group were excluded due to miniscrew failure. Hence, 23 participants completed the study, 12 in the piezocision group, and 11 in the control group.

**Fig. 1:**
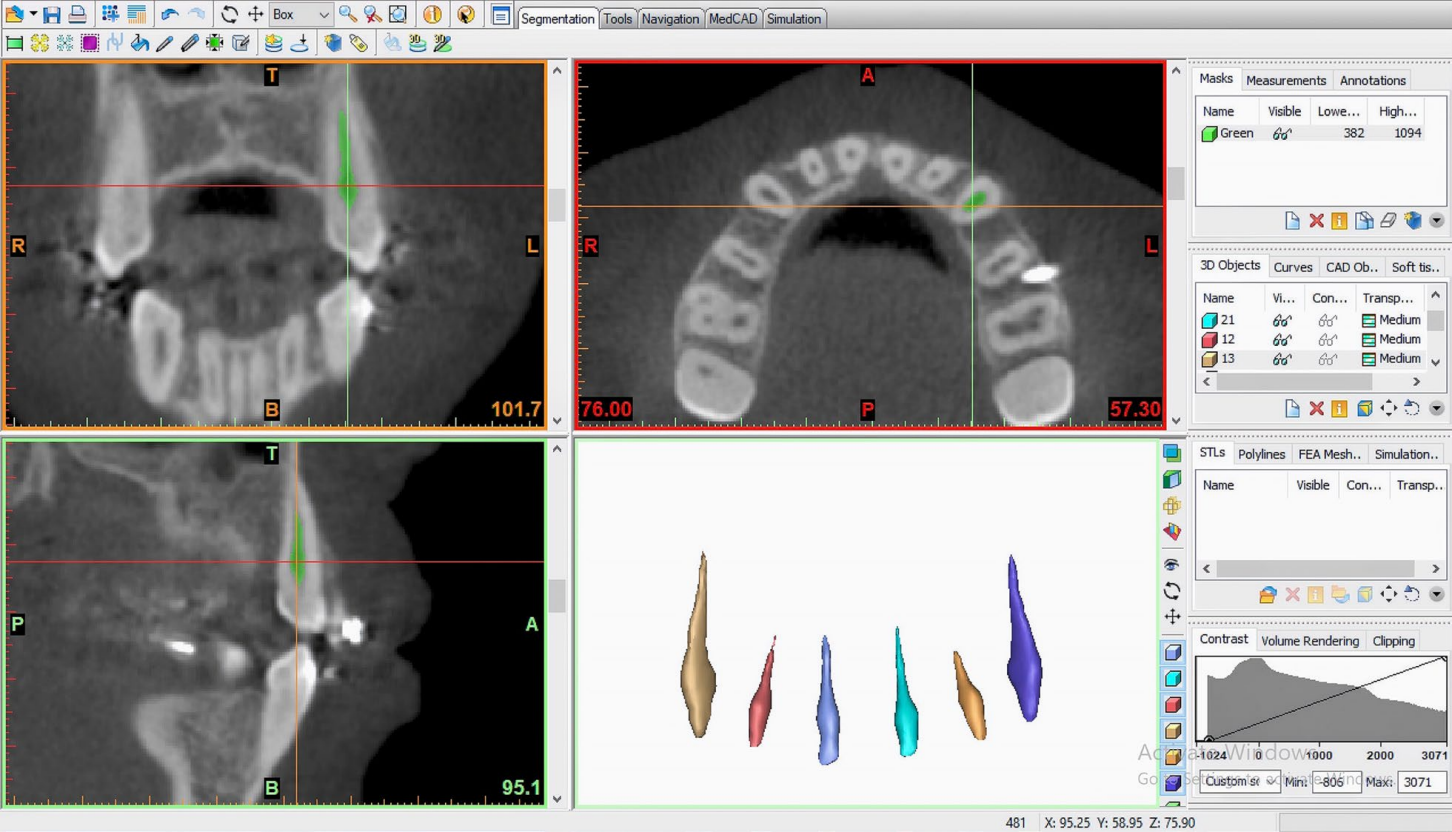
Screenshot image for one of the included patients illustrating the segmented pulp tissue, from the CBCT scan, in all 3 planes of space and source of 3-Dimensional images of the pulp cavities for the maxillary anterior teeth (image rendered using Mimics imaging software)

CBCT images were acquired using the i-CAT next generation CBCT machine (I-CAT Cone Beam 3D Imaging, Imaging Sciences International Hatfield, PA, USA). CBCT images were taken during the initial orthodontic records visit (T0) and at the end of en-masse retraction, mean = 122.74 ± 3.06 days (T1). Pulp volume of each of maxillary anterior teeth (canine to canine) was measured by one blinded investigator using an image processing software (Mimics Version 14 on Windows; Materialize, Leuven, Belgium). Pulp volume measurements were performed by segmenting and separating the images of the selected teeth (maxillary central and lateral incisors and canines) which were carried out by grayscale threshold as previously described [8]. Briefly, using the axial, sagittal, and coronal views, a mask was built and cropped in these 3 axes to segment the pulp cavity of the selected tooth, then an optimum separating grayscale threshold was selected. The threshold values were established separately with the same Hounsfield Units (HU) for each tooth at T0 and T1, then the calculation of the 3-dimensional image was performed. The mask of the pulp cavity in 3-dimensional allowed the operator to calculate the pulp volume (Fig. 1). The root lengths of each maxillary six anterior teeth were measured along the long axis of the teeth using the CBCT frontal view. The measurements were recorded from the middle of the cementoenamel junction to the root apex.

Intra-examiner reliability was tested using the intra-class correlation coefficient (ICC). Measurements of 10 participants were repeated after 2 weeks to assess measurement errors. The ICC coefficient ranged between 0.79 and 0.98%, indicating excellent reliability.

## Statistical analysis

Descriptive statistics (means and standard deviations) were calculated. The normality of the main dependent variables tested using the Shapiro-Wilks test (*P* > 0.05) showed that they were almost normally distributed in both groups. Paired t-tests were used to compare mean changes (T0 vs T1) within groups and independent sample *t*-tests were used to compare mean differences (T0 − T1) between groups. Chi-square was used to test the distribution of males and females between and within groups. Pearson’s correlation coefficient was used to assess the relationship between the mean changes of each tooth pulp volume and its root length. Data were analyzed using the Statistical Package for Social Sciences (IBM SPSS Statistics for Macintosh, Version 26.0. Armonk, NY, USA). The significance level was set at α = 0.05 by a statistician who was blinded to the results.

## Results

**Fig. 2:**
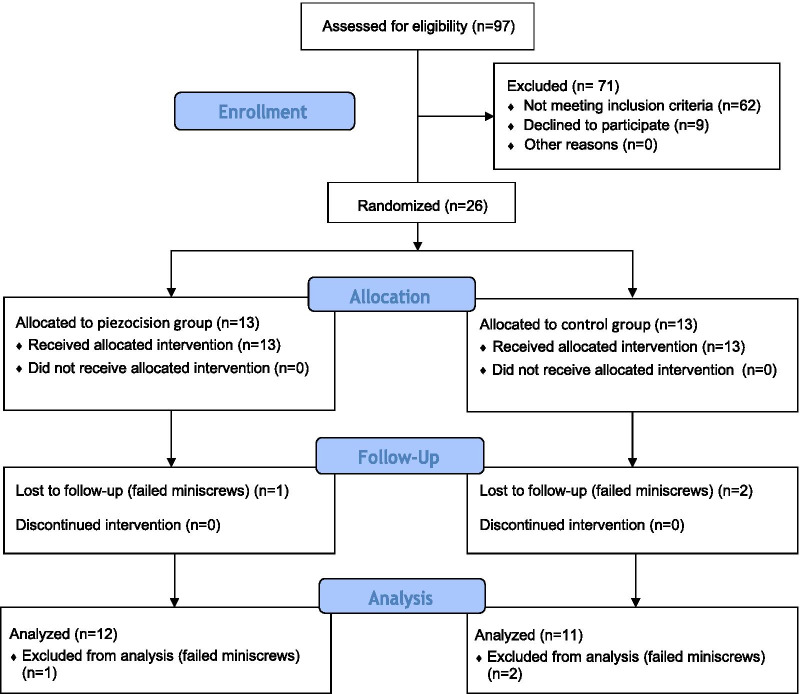
CONSORT flow diagram

A total of 26 participants were included in this study (Fig. 2), 13 in the piezocision group (7 males, 6 females) and 13 in the control group (6 males, 7 females). The enrollment began in September 2016 and completed in January 2018. from the 26 patients enrolled, one patient in the piezocision group, and two patients in the control group were eliminated after enrollment due to miniscrews failure. At the commencement of the trial the mean age of the patients allocated to the piezocision group was 19.27 (± 3.38) years while the mean age of the control group was 20.83 (± 3.64) years.

Descriptive statistics and comparisons between both groups are presented in Table 1. There was no statistically significant difference in age between the groups (*P* = 0.30). The chi-square test showed that there was equal gender distribution in both groups (*P* = 0.99). Also, all initial measurements (T0) were not significantly different between both groups.

**Table 1:** Descriptive summary of initial measurements (T0) and intergroup comparisons of pulp volume and gender distribution

Variables	Piezocision group (n = 12)	Control group (n = 11)	*p* value
Age (years)	19.27 ± 3.38	20.83 ± 3.64	0.30
Gender [n (%)]	Male = 6 (55%) Female = 6 (50%)	Male = 5 (45%) Female = 6 (50%)	0.99*
Pulp Volume (mm^3^)			
Right central	31.71 ± 9.67	33.15 ± 12.95	0.76
Right lateral	23.72 ± 6.03	23.24 ± 10.86	0.90
Right canine	46.84 ± 16.42	45.26 ± 14.53	0.81
Left central	31.22 ± 9.65	32.56 ± 10.90	0.76
Left lateral	22.28 ± 7.17	23.73 ± 11.63	0.72
Left canine	46.11 ± 14.04	48.40 ± 15.34	0.71

Data are presented as mean ± SD, Gender is presented as number (%), * Chi square test

Table 2 shows the comparison of pulp volume means within and between the piezocision and control groups. There was a statistically significant decrease in pulp volume at the end of en-masse retraction in both groups for all anterior teeth (*P* < 0.01). When comparing the mean change in pulp volume in the piezocision and control groups, no significant differences were observed, *P* > 0.05 (Table 2).

**Table 2:** Comparisons of pulp volume means within and between the Piezocision and Control groups

Pulp volume (mm^3^)	Piezocision group (n = 12)			T0 versus T1	Control group (n = 11)			T0 versus T1	Piezocision versus control
	T0	T1	Mean diff	*P value*	T0	T1	Mean diff	*P value*	*P value*
Right central	31.71 ± 9.67	30.79 ± 9.64	0.92 ± 0.82	0.003	33.15 ± 12.95	32.19 ± 12.89	0.97 ± 0.42	< 0.001	0.87
Right lateral	23.72 ± 6.03	22.34 ± 6.42	1.38 ± 0.97	< 0.001	23.24 ± 10.86	22.16 ± 11.01	1.09 ± 0.68	< 0.001	0.40
Right canine	46.84 ± 16.42	45.77 ± 16.41	1.08 ± 0.55	< 0.001	45.26 ± 14.53	44.01 ± 14.48	1.25 ± 0.84	0.001	0.56
Left central	31.22 ± 9.65	30.37 ± 9.58	0.84 ± 0.51	< 0.001	32.56 ± 10.90	31.30 ± 10.85	1.25 ± 0.71	< 0.001	0.13
Left lateral	22.28 ± 7.17	21.26 ± 6.99	1.02 ± 0.78	0.001	23.73 ± 11.63	22.82 ± 11.43	0.90 ± 0.43	< 0.001	0.67
Left canine	46.11 ± 14.04	45.02 ± 13.88	1.09 ± 0.74	< 0.001	48.40 ± 15.34	47.47 ± 15.30	0.93 ± 0.64	0.001	0.59

Data are presented as mean ± SD**,** T0 = initial, T1 = end of en masse retraction

As shown in Table 3, there was a statistically significant root resorption at the end of en-masse retraction in both the piezocision and control group. Only the right and left central incisors and right canine showed statistically significantly more root resorption in the control group compared to the piezocision group (0.09 ± 0.39, 1.00 ± 0.53, and 1.03 ± 0.63, respectively), *P* < 0.05.

**Table 3:** Comparisons of root length means within and between the Piezocision and Control groups

Root length	Piezocision group (n = 12)			T0 versus T1	Control group (n = 11)			T0 versus T1	Piezocision versus control
	T0	T1	Mean diff	*p value*	T0	T1	Mean diff	*p value*	*p value*
Right central	25.04 ± 1.04	24.47 ± 1.17	0.58 ± 0.27	< 0.001	25.06 ± 1.41	24.16 ± 1.47	0.90 ± 0.39	< 0.001	0.030
Right lateral	22.88 ± 0.75	22.44 ± 0.81	0.44 ± 0.30	< 0.001	23.38 ± 1.53	22.56 ± 1.39	0.82 ± 0.60	0.001	0.065
Right canine	27.28 ± 1.29	26.69 ± 1.41	0.58 ± 0.27	< 0.001	27.68 ± 2.16	26.68 ± 1.97	1.00 ± 0.53	< 0.001	0.025
Left central	25.23 ± 0.98	24.63 ± 0.93	0.60 ± 0.26	< 0.001	25.25 ± 1.33	24.22 ± 1.43	1.03 ± 0.63	< 0.001	0.046
Left lateral	22.91 ± 0.77	22.28 ± 0.81	0.63 ± 0.25	< 0.001	23.53 ± 1.69	22.70 ± 1.54	0.83 ± 0.34	< 0.001	0.140
Left canine	27.14 ± 1.12	26.50 ± 1.12	0.64 ± 0.26	< 0.001	27.47 ± 1.79	26.66 ± 1.64	0.81 ± 0.30	< 0.001	0.163

Data are presented as mean ± SD**,** T0 = initial, T1 = end of en masse retraction,

Pearson’s correlation coefficient was calculated between the mean changes of each tooth pulp volume and its root length (Table 4). There was only a statistically significant but moderate correlation in the maxillary right canine, r = 0.44, *P* = 0.034. No significant correlations were observed between the pulp volume of the remaining teeth and their root lengths (*P* > 0.05).

**Table 4:** Bivariate correlations between pulp volume and root resorption

	Root resorption					
	Central right	Lateral right	Canine right	Central left	Lateral left	Canine left
*Pulp volume*						
Central right	0.14	0.28	0.20	0.14	0.25	− 0.01
	(0.51)	(0.20)	(0.35)	(0.51)	(0.25)	(0.96)
Lateral right	− 0.05	0.18	− 0.03	− 0.05	− 0.17	− 0.04
	(0.82)	(0.42)	(0.91)	(0.82)	(0.45)	(0.85)
Canine right	0.15	0.09	**0.44**	0.15	0.41	0.09
	(0.50)	(0.69)	**(0.03)**	(0.50)	(0.05)	(0.70)
Central left	− 0.03	**0.47**	0.21	− 0.03	0.13	− 0.11
	(0.90)	**(0.03)**	(0.34)	(0.90)	(0.57)	(0.62)
Lateral left	− 0.13	0.02	− 0.22	− 0.13	− 0.37	− 0.15
	(0.55)	(0.91)	(0.31)	(0.55)	(0.08)	(0.51)
Canine left	− 0.21	0.26	− 0.22	− 0.21	− 0.14	− 0.16
	(0.34)	(0.24)	(0.31)	(0.34)	(0.53)	(0.46)

Data are presented as correlation coefficient “r” and (*p* value)

## Discussion

The cause and effect relationship between orthodontic tooth movement and dental pulp volume and root resorption is still a challenging phenomenon [1–12, 21, 22]. It is well recognized that orthodontic forces do affect teeth in many aspects including the periodontium, root length, and pulp structure [16, 23, 24]. Dental pulp was found to react to orthodontic forces in different ways, but in general, pulp responds by forming tertiary dentin after its irritation [25]. These types of repair could adversely affect the volume of the pulp. Multiple studies tested the effect of orthodontic tooth movements by using, for example, histological assessment using light or scanning electron microscopy and 2-dimensional radiograph [3, 14, 26–28]. These methods, however, have a limitation that could reduce their accuracy. The 3-dimensional measuring technique used in the current study provided an accurate method for the evaluation of the pulp volume during orthodontic treatment. In one study, 3-dimensional radiography was used to assess pulp volume changes concurrent with orthodontic treatment [8]. Pulp volume of the anterior teeth was found to undergo significant reduction after orthodontic treatment. They attributed their findings to the protective reaction of the pulp to the irritation caused by orthodontic teeth movements, which led to the formation of tertiary dentin that restricted the available pulp volume [8]. They concluded that the pulp volume undergoes a significant reduction in the anterior teeth after orthodontic treatment. This reduction of the pulp volume could be explained by the possible alterations in the pulpal blood supply and inflammation caused by orthodontic teeth movement that may have led to a mild irritation to odontoblastic layer surrounding the pulp and the release of inflammatory mediators that stimulates odontoblast and odontoblast-like cells, leading to the deposition of tertiary dentin with the eventual reduction in pulp space [29, 30].

In our study, the percentage of pulp volume reduction was 3.4% in the piezocision group and 2.7% in the control group. These percentages are not consistent with Venkatesh et al.[8]. This difference could be conjectured by the shorter duration of our study which did not allow sufficient time for secondary dentine to form [25, 30]. The time limit in our study was selected based on the fact that the effect of the regional acceleratory phenomenon peaks at 1 to 2 months, and weakens after 4 to 6 months [31]. Another explanation for the different results could be due to the mechanics used in our study during tooth movement. The current study also found that the change in pulp volume was not related to root resorption and that both responses could be mutually independent. Since there are no other similar studies, comparison to our findings is difficult to conduct.

A limitation of the current study was the small sample size as the sample was taken from a previous study [19]. Still, the sample size is similar to previous root resorption and pulp volume studies [9, 32]. Another limitation is that it cannot be used to predict the long-term effect of piezocision-assisted orthodontic tooth movement on the pulp volume. Furthermore, since this was a radiographical study, the nature of pulp volume reduction cannot be determined. In addition, histological changes in the pulp cannot be assessed by this technique. Future histological studies to evaluate the biological mechanism with larger samples are warranted. Moreover, this study was limited to the maxilla future study are required to evaluate change of pulp volume in both jaws.

## Conclusion

The effect of piezocision-assisted orthodontic tooth movement on the pulp volume was comparable to the conventional orthodontic treatment. The degree of change in pulp volume does not appear to be related to the amount of root resorption. Future studies with a larger sample size are needed to evaluate the long-term effect of piezocision-assisted orthodontic tooth movement.

## Data Availability

The datasets used and analyzed during the current study are available from the corresponding authors upon reasonable request.

## Abbreviations

CBCT: Cone-beam computed tomography
T0: Initial orthodontic records visit
T1: End of en-masse retraction
HU: Hounsfield Units
ICC: Intra-class correlation coefficient
SPSS: Statistical Package for Social Sciences
